# Improving quality and assessment of referrals to the Enfield Crisis Resolution and Home Treatment Team (ECRHTT)

**DOI:** 10.1192/bjo.2021.541

**Published:** 2021-06-18

**Authors:** Margarita Kousteni, John Cousins, Ajay Mansingh, Maja Elia, Yumnah Ras, Mercedes Chavarri, Marilia Gougoulaki, Imo Akande

**Affiliations:** Barnet Enfield and Haringey Mental Health NHS Trust

## Abstract

**Aims:**

Triaging referrals to crisis resolution and home treatment teams is a significant undertaking requiring experienced and dedicated staff. We observed that the volume of inappropriate referrals to ECRHTT was high, and that staff processing these often felt inexperienced or lacking in confidence to discharge them back to the referrers and signpost them to appropriate services.

The aims of this quality improvement project (QIP) were:

- to reduce the number of inappropriate referrals received by the team
- to reduce the number of inappropriate referrals accepted by the team

This would significantly improve access and flow to the service and facilitate better patient care.

**Method:**

A pilot study was first completed of the quality (appropriateness/ inappropriateness) and source of all referrals to ECRHTT in January 2019 (n = 177).

Subsequently, the consultant psychiatrist for ECRHTT based himself within the assessment team. He was able to closely monitor the referrals, at the same time as providing medical input to patients at their first point of contact. To evaluate the impact of this intervention, the percentage of inappropriate referrals accepted pre- and post-change was compared by re-auditing all referrals received in February 2019 (n = 175).

Further interventions were instigated to improve referral quality. These included continuation of psychiatric medical input to the assessment team, teaching sessions for GPs and the crisis telephone service, and weekly meetings with psychiatric liaison and community mental health teams (CMHTs). Change was measured by reassessing the quality of all referrals made to ECRHTT in February 2020 (n = 215).

**Result:**

46.9% of inappropriate referrals to ECRHTT were accepted in January 2019 compared to 16.9% in February 2019 following the addition of medical input to the assessment team. The absolute difference was 30% (95% CI: 14%–44%, p < 0.001).

71% of referrals from GPs were inappropriate in January 2019 compared to 36% in February 2020 post-intervention (difference 35%, 95% CI: 8.84%–55.4%, p < 0.05). Inappropriate referrals from CMHTs decreased from 55.5% to 12% (difference 43.5%, 95% CI: 9.5%–70.3%, p < 0.05). Overall, the percentage of inappropriate referrals fell from 38% to 27.4%, a difference of 10.6% (95% CI: 1.3%–19.8%, p < 0.05). The percentage of inappropriate referrals from liaison teams did not change significantly.

**Conclusion:**

This piece of work shows that better engagement with referral sources significantly improved the quality of referrals made to ECRHTT. Interventions included medical input at the point of referral, teaching sessions for general practitioners as well as ongoing liaison with referring teams.

